# Hepatotoxicity or Hepatoprotection? Pattern Recognition for the Paradoxical Effect of the Chinese Herb *Rheum palmatum* L. in Treating Rat Liver Injury

**DOI:** 10.1371/journal.pone.0024498

**Published:** 2011-09-06

**Authors:** Jia-bo Wang, Hai-ping Zhao, Yan-ling Zhao, Cheng Jin, Dao-jian Liu, Wei-jun Kong, Fang Fang, Lin Zhang, Hong-juan Wang, Xiao-he Xiao

**Affiliations:** 1 Integrative Medicine Center, 302 Military Hospital, Beijing, People's Republic of China; 2 Key Laboratory of Modern Preparation of Traditional Chinese Medicine, Ministry of Education, Jiangxi University of Traditional Chinese Medicine, Nanchang, People's Republic of China; 3 Chengdu University of Traditional Chinese Medicine, Chengdu, People's Republic of China; Saint Louis University, United States of America

## Abstract

The hepatotoxicity of some Chinese herbs has been a cause for concern in recent years. However, some herbs, such as rhubarb, have been documented as having both therapeutic and toxic effects on the liver, leading to the complex problem of distinguishing the benefits from the risks of using this herb. To comparatively analyze the dose-response relationship between rhubarb and hepatic health, we administrated total rhubarb extract(RE) to normal and carbon tetrachloride(CCl_4_)-treated rats for 12 weeks at 4 dosage levels(2.00, 5.40, 14.69 and 40.00 g·kg^−1^, measured as the quantity of crude material), followed by biochemical and histopathological tests of the rats' livers. A composite pattern was extracted by factor analysis, using all the biochemical indices as variables, into a visual representation of two mathematically obtained factors, which could be interpreted as the fibrosis factor and the cellular injury factor, according to the values of the variable loadings. The curative effect of administering the two lowest dosages of RE to CCl_4_-treated rats was mainly expressed as a decrease in the extent of cellular injury. The hepatoprotective mechanism of RE might be related to its antioxidant effect, the antagonism of the free radical damage to hepatocytes caused by CCl_4_. By contrast, the RE-induced liver damage was mainly expressed as a significant increase in the amount of fibrosis in both normal rats at all dosage levels and CCl_4_-treated rats at the two highest dosage levels. Therefore, the hepatotoxic potential of RE could be attributable to the liver cell fibrosis induced by high doses of the herb. This study illustrates the bidirectional potential of rhubarb and demonstrates the feasibility of using factor analysis to study the dose-response relationships between herbal medicines and hepatotoxicity or the healing effects of these herbs by extracting the underlying interrelationships among a number of functional bio-indices in a holistic manner.

## Introduction

Traditional Chinese herbal medicines are used worldwide, but there are a number of concerns regarding their use. Some researchers believe that herbal medicines are effective and safe, while others doubt their effectiveness and safety. The latter group has increased in number, especially after consecutive reports of adverse hepatotoxic effects of a number of phytomedicines, including *Polygonum multiflorum* [1], Shou-Wu Pian [2], Xiao-Chai-Hu-Tang (Syo-saiko-to), [3] and some herbs containing anthraquinones, such as *Sennae fructus angustifoliae* [4] and *Rheum palmatum* L. (rhubarb) [5]. Chitturi et al. believe that herbal hepatotoxicity is an expanding but poorly defined problem [6].

These herbal medicines, especially rhubarb and its constituent components, are frequently used as treatments for chronic liver diseases and as laxatives in Asian countries, and a number of cases have demonstrated their affirmative effectiveness and safety [7], [8]. In addition to the clinical evidence of the effectiveness of rhubarb in treating chronic hepatic diseases, [9], [10] many experiments at the animal, cellular and molecular levels have revealed that rhubarb and its anthraquinone constituents exert a protective effect against hepatic injury by inhibiting the carbon tetrachloride (CCl_4_)-induced elevation of serum alanine aminotransferase (ALT), aspartate aminotransferase (AST), hyaluronic acid (HA) and laminin (LN) levels. [11], [12]

In vitro and in vivo studies focusing on the anthraquinone constituents of rhubarb, however, have revealed conflicting results. A two-year National Toxicology Program (NTP) study, conducted by the U.S. Department of Health and Human Services (DHHS) on emodin, one of the main anthraquinone derivatives contained within rhubarb, demonstrated the hepatotoxic potential of emodin on normal rats and mice. The toxic effects included elevations of ALT levels, total bile acids (TBIL) levels and organ-weight-to-body-weight ratios (relative weights) of the liver, as well as decreases in alkaline phosphatase (AP) and total protein levels (TP). [13] Other investigators have reported that administration of rhubarb extracts (REs) led to elevations of serum ALT and TBIL levels in patients and experimental animals, leading to hepatitis and nephritis. [14]–[17]

The conflicting results of these reports might be due to the heterogeneity and complexity of herbal medicines with respect to their geographic origin, harvest season, and discrepancy of constituent components. Herbal medicines, which usually contain hundreds of chemical components with broad pharmacological targets and effects, present difficulties in describing overall biological profiles in a simple manner with a few essential factors that can be readily comprehended. Many phytopharmacologists have dedicated themselves to solving this provlem for decades. In recent years, pattern recognition, [18] a type of multivariate analysis approach, has become a promising method for identifying and interpreting meaningful regularities in noisy or complex systems, by analyzing data from different perspectives and summarizing them as useful information or by mining potential interrelationships. The factor analysis used in this study is a common pattern recognition method that reduces a large number of disease parameters to a relatively small number of independent factors, by describing the covariance structure of the observed data variables in terms of a few underlying and independent but non-measurable features called “factors”. [19]

The aim of this study was to determine the paradoxical effects of liver protection and hepatotoxicity of rhubarb in the treatment of rat experimental hepatitis induced by carbon tetrachloride by categorizing a number of functional bio-indices into separate complementary domains (protection or toxicity) with no *a priori* assumptions. We believe that our data will provide useful information for the safe evaluation and proper application of rhubarb and, furthermore, will help increase our understanding of the rational use of phytomedicines.

## Materials and Methods

### Ethics Statement

This study was conducted in strict accordance with the recommendations of the Guidelines for the Care and Use of Laboratory Animals of the Ministry of Science and Technology of China. The animal protocol was approved by the Committee on the Ethics of Animal Experiments of the 302 Military Hospital (Approval ID: 09-056).

### Animals and reagents

Male and female Sprague- Dawley rats, aged 6-8 weeks and weighing 180 ± 30 g, were obtained from the Laboratory Animal Center of the Academy of Military Medical Sciences (License No. SYXK 2007- 004). The animals were raised separately by gender and had unlimited access to food and water in an environmentally controlled breeding room (temperature 22±2°C, humidity 60–80%). The breeding room was illuminated by an artificial light cycle with 12 hours of light and 12 hours of darkness every day and was disinfected regularly.

Carbon tetrachloride (CCl_4_) was purchased from Beijing Beihua Fine Chemicals Co. Ltd (Cat. #20060525), and olive oil was purchased from Sinopharm Chemical Reagent Beijing Co. Ltd (Cat. #F20080219). Both reagents were of AR grade.

### Herb extraction and UPLC-MS/MS analysis

Rhubarb is the peeled and dried root of *Rheum palmatum* L., *R. tanguticum Maxim. ex* Balf. or *R. officinale* Baill. (Polygonaceae family), as stipulated in the Chinese Pharmacopoeia[20]. Rhubarb is also officially listed in European and Japanese pharmacopoeias. [21], [22] *R. palmatum* was used in this study because it is described in all three pharmacopoeias and is more commonly used than the other species. The dried root and rhizome of *R. palmatum* L. were collected in Lixian County of the Gansu Province of China and were identified by Professor Xiao-he Xiao, a taxonomist at the Military Institute of Chinese Materia Medica. In total, 100 kg rhubarb was added to 600 L of 90% ethanol and then heated and extracted 3 times for 1 hour each time. The residual compounds after extraction were added to 1000 L of water and then heated and extracted for 1 hour. The filtrate was merged and sprayed dry, producing a yield of 29.3%. The samples were preserved for further experiments.

Rhubarb extract (RE) was analyzed for quality control by ultra-performance liquid chromatography coupled with an ultraviolet-visible detector and electrospray ionization tandem mass spectrometry (UPLC-UV-ESI-MS/MS). [23] The experimental conditions and data are provided in Text S1.

### Animal model and study groups

In this study, a CCl_4_-induced liver injury model was used because it has been well documented and demonstrated to have good repeatability and reliability. One experiment cycle continued for 16 weeks, and the following procedure was used immediately prior to the experiment, 180 rats were randomized into 10 groups of 18 animals each, with equal distributions by gender. The groups shown in Tables 1 and 2 were differentiated based on the treatment group and extract dosage. The rats in the five model groups (groups M and M_1_-M_4_) were injected intraperitoneally (i.p.) with CCl_4_ oil (containing 1 portion CCl_4_ and 9 portions olive oil, 5 ml/kg) 2 times per week for 12 weeks to induce chronic liver injury, and the rats in the other groups received normal saline (N.S.). The rats in all medication groups (groups N_1_-N_4_ and M_1_-M_4_) were administered RE intragastrically once per day from the 4^th^ week until the end of the 16^th^ week. Rats in the control (group N) and model (group M) groups were administered normal saline intragastrically. The RE dosages were 2.00, 5.40, 14.69 and 40.00 g/kg (measured as the quantity of crude material) per kg of body weight once per day and were equivalent to between 4 and 80 times the human upper dosage limit stipulated in the Chinese Pharmacopoeia (0.5 g/kg). Food and water were available *ad libitum* for all rats throughout the experiment.

**Table 1:** Liver toxicity induced by rhubarb extract in normal rats with regard to survival and biochemical indices.

Groups	N	ND_1_	ND_2_	ND_3_	ND_4_
Dosage (g/kg)	0	2	5.4	14.69	40
Survival^a^	18	18	17	15	14
ALT (U/L)	73.00±17.70	104.86±10.17	119.43±31.36△	125.43±19.86△	175.86±22.75△
AST (U/L)	149.57±27.78	187.00±37.46	207.86±71.81	268.00±57.83△	261.00±48.55△
AP (U/L)	53.71±21.26	73.00±28.14	89.29±18.31	97.29±21.48△	116.43±19.7△
TBIL (μmol/L)	1.09±0.32	1.01±0.59	1.24±0.30	1.50±0.53	1.66±0.75△
TP (g/L)	69.71±12.31	74.14±13.56	76.29±11.34	84.43±10.72	87.71±8.12△
GLO (g/L)	27.57±7.50	34.43±6.48	35.71±5.77	46.29±9.72	51.43±7.57△
HA (ng/L)	203.30±71.98	220.4±73.97	277.79±23.17△	281.46±44.08△	350.13±47.98△
LN (ng/L)	31.53±5.05	36.76±10.47	38.01±8.43	48.47±10.22△	54.19±9.78△
TGF-β_1_ (pg/L)	18.23±1.42	20.87±1.95	19.09±3.38	29.82±6.27	39.19±7.76△

N, normal rat group without treatment; ND_1_-ND_4_, normal rat groups treated with rhubarb extract at dosages of 2.00, 5.40, 14.69 and 40.00 g kg^−1^, respectively.

ALT, serum alanine aminotransferase; AST, serum aspartate aminotransferase; AP, alkaline phosphatase; TBIL, total bile acids; TP, total protein; GLO, globulin; HA, hyaluronic acid; LN, laminin; TGF-β_1_, transforming growth factor β_1_.

a The initial number of animals in each group of the study was 18.

△ *P<0.05* compared to the N group.

**Table 2:** Protective effect of rhubarb extract against CCl_4_-induced liver injury with regard to the survival and biochemical indices in the model groups.

Groups	M	MD_1_	MD_2_	MD_3_	MD_4_
Dosage (g/kg)	0	2	5.4	14.69	40
Survival^a^	16	18	15	13	11
ALT (U/L)	519.14±64.46△	261.14±46.52*	213.43±33.16*	183.14±33.26*	253.43±55.24*
AST (U/L)	775.14±119.95△	421.00±35.13*	234.71±43.18*	268.57±77.34*	367.57±104.59*
AP (U/L)	140.57±58.8△	58.86±15.55*	63.57±20.56*	83.00±18.35	103.57±27.67
TBIL (μmol/L)	2.34±0.24△	1.49±0.71*	1.24±0.72*	1.64±1.07	2.89±0.72*
TP (g/L)	92.43±14.12△	71.00±10.85*	71.00±6.68*	75.71±9.41	87.14±7.99
GLO (g/L)	58.43±9.36△	29.86±10.93*	33.00±3.51*	38.14±8.28	49.71±6.70
HA (ng/L)	362.17±36.88△	284.23±38.73*	310.29±70.63*	436.37±61.71*	508.10±62.38*
LN (ng/L)	53.31±23.25△	36.59±10.25	38.64±7.85	52.49±6.93	91.96±19.21*
TGF-β_1_ (pg/L)	33.07±11.06△	30.69±6.75	32.48±5.73	34.08±6.71	53.77±10.45*

M, model rat group without treatment; MD_1_-MD_4_, model rat groups treated with rhubarb extract at dosages of 2.00, 5.40, 14.69 and 40.00 g kg^−1^, respectively.

ALT, serum alanine aminotransferase; AST, serum aspartate aminotransferase; AP, alkaline phosphatase; TBIL, total bile acids; TP, total protein; GLO, globulin; HA, hyaluronic acid; LN, laminin; TGF-β_1_, transforming growth factor β_1_.

a The initial number of animals in each group of the study was 18.

△ *P<0.05* compared to the N group.

*P<0.05, compared to the M group.

### Histopathologic examination and biochemical analysis

Necropsies were performed on all the animals at the end of the experiment. Tissues for histopathologic examination were fixed and preserved in 10% neutral buffered formalin, processed and trimmed, embedded in paraffin, sectioned to a thickness of approximately 5μm, and stained with hematoxylin and eosin (HE).

Biochemical analyses were performed on all animals. The biochemical parameters of serum ALT, AST, AP, urea nitrogen (SUN), creatinine (CREA), TP, albumin (ALB), globulin (GLO), calcium, inorganic phosphate, total cholesterol (CHOL) and TBIL levels were measured by the Clinical Laboratory of 302 Military Hospital of China. The levels of HA, LN and transforming growth factor β_1_ (TGF-β_1_) in rat serum were quantitated by chemiluminescence using assay kits from Beijing Deyuan Biomedical Engineering Corporation, China.

### Statistical analysis

The experimental values were expressed as means ± standard deviation (means ± SD). All data were analyzed using the Statistical Package for the Social Sciences for Windows, version 13.0 (SPSS Inc, Chicago, IL, USA). The groups were compared using ANOVA and multivariate statistical analysis, and the significance probability was set at *P* = 0.05.

The factor analysis model was as follows:
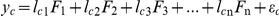
where *y_c_* is the *c*th original variable, *c* = 1, . . . , *p*, *p* is the number of original variables, *l_c_*_1_, *l_c_*_2_, *l_c_*_3_, . . . , *l_c_*_n_ are the factor loadings, *F*_1_, *F*_2_, *F*_3_, . . . , *F*_n_ are the (new) factors, *n* is the number of factors, and *ε_c_* the error term related to *y_c_*. [24] The correlation coefficients were analyzed by principal component analysis and subsequent rotation according to the standard varimax criterion. The correlation between parameters was attributed to their common dependence on independent entities called “factors.” The coefficients that linked the parameters to factors were named “factor loadings”.

## Results

### Study design

This study included two parts. First, a comparative experiment was designed to investigate the dose-responses of RE to normal and CCl_4_-treated rats. Standard blood chemistry analyses were used to evaluate liver damage and histological changes were assessed. All doses were set at high levels to uncover potential toxicities to rigorously investigate the hepatic risks of RE.

Second, a factor analysis approach was utilized to mathematically illustrate the bidirectional effect of liver protection and hepatotoxicity of RE on subjects under different physiological conditions. The mathematical modeling process used a dimension reduction effect to convert the original dataset of numerous disease variables into a relatively small number of independent “factors” that are not measurable but are easy to comprehend, including the protection factor and the lesion factor.

### Identification of the chemical constituents in rhubarb extract (RE)

**Figure 1:**
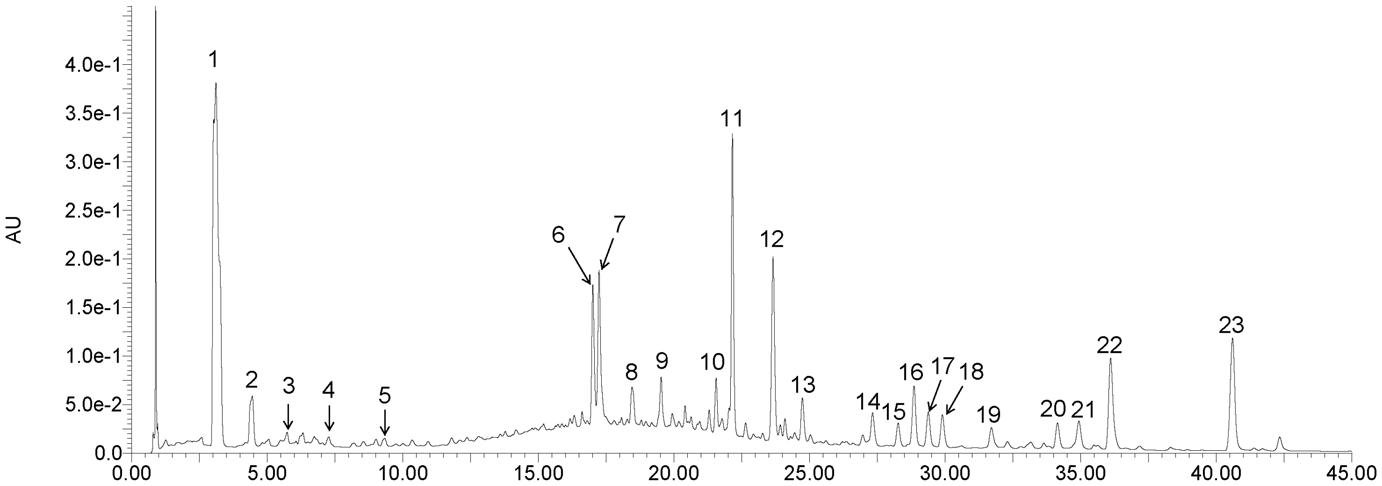
UPLC/UV chromatogram at 280 nm of the extract of *Rheum palmatum* L. Seventeen compounds were identified by electrospray ionization tandem mass spectrometry: galloyl glucose (1), glucopyranosyl-galloyl-glucose (3), catechin glucopyranoside (4), isomer of catechin glucopyranoside (5), aloe emodin *O*-glucoside (9), rhein *O*-glucoside (10), cinnamoyl-*O*-galloyl-glucose (11), laccaic acid D *O*-glucoside (13), dehydroxylaccaic acid D *O*-glucoside (14), chrysophanol *O*-glucoside (15), isomer of chrysophanol *O*-glucoside (16), emodin *O*-glucoside (17), laccaic acid D (19), physcion *O*-glucoside (20), emodin (21), acetyl- chrysophanol (22) and physcion (23). The other six chromatographic peaks (2, 6-8, 12 and 18) are unknown compounds.

In a previous study, it was demonstrated that the major components contained in RE are anthraquinone derivatives and tannin-related compounds. [25] Using liquid chromatography coupled with tandem mass spectrometry, seventeen compounds were identified in this study, and their structures were tentatively assigned as twelve anthraquinone derivatives and five tannin-related compounds. The identified chemical constituents (Table S1) and the contents of the index compounds in the RE (Table S2) are summarized in the supplementary materials to illustrate the chemical basis of the RE utilized in this study. The chemical structures of the identified constituents in RE are depicted in Figures S1 and S2. The chromatographic profile is depicted in Fig. 1.

### Common changes

All the RE-treated rats had a dose-related fur color change patterns, ranging from yellow to red, that appeared early in the study with exposure to increasing concentrations of RE. Red-to-brown urine and reddish-brown staining of the anal area were also noted for all rats that were treated with RE. Marked and chronic diarrhea accompanied by emaciation and inactivity were observed in all model and normal rats that were treated with 40 g/kg, but not the lower doses, of RE. During RE administration, the rats in the CCl_4_-treated groups had lower increases in weight than those in the normal groups at the same dosage. Rats in all the RE-administered groups had negative dose-related body weight changes, and the rats in the highest dosage group had the lowest increase in weight until cessation of RE administration.

### Histopathology

Dosage-dependent vacuolar degeneration of the hepatic portal area and/or hyperplasia of interstitial fibrous tissue were observed in over 67% of animals in the ND_1_-ND_4_ groups.

**Figure 2:**
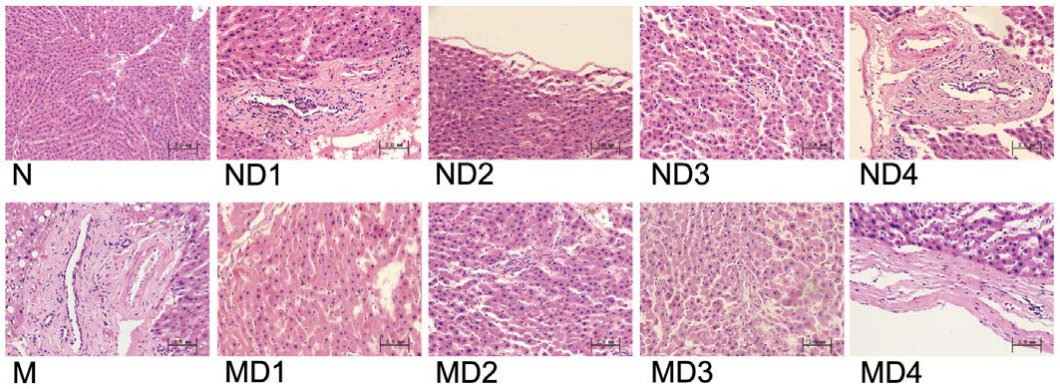
Typical histopathologic section photographs of rats in different groups (HE stained, 200× magnification). Dose-dependent vacuolar degeneration of the portal area and/or hyperplasia of the interstitial fibrous tissue were observed in the normal groups ND_1_–ND_4_. Obviously thickened envelopes and fibrosis of the portal area and/or stroma was observed in the animals of group M. This toxic degeneration caused by CCl_4_ was improved after treatment with rhubarb extract in groups MD_1_–MD_4_. Notably, alleviation of fibrosis of the liver envelope, portal area, and/or stroma was observed in the animals in the MD_1_ and MD_2_ groups, whereas aggravation of fibrosis was found in over 67% of animals in the MD_3_ and MD_4_ groups. N, normal rat group without treatment; ND_1_-ND_4_, normal rat groups treated with rhubarb extract at dosages of 2.00, 5.40, 14.69 and 40.00 g kg^−1^, respectively. M, model rat group without treatment; MD_1_-MD_4_, model rats groups treated with rhubarb extract at dosages of 2.00, 5.40, 14.69 and 40.00 g kg^−1^, respectively.

In the control group (group N), all rats exhibited normal histology. By contrast, 16 out of 18 animals in the model group (group M) exhibited vacuolar degeneration, which was characterized by small or medium vacuole formation in hepatocytes accompanied by nuclear loss or dislocation to the margins of the vacuoles. Hepatocytes with large nuclei were occasionally observed. Small focal lymphocytic infiltration of the stroma and/or portal area was also observed in 12 out of 18 animals in group M. Moreover, an obviously thickened envelope and fibrosis of the portal area and/or stroma were observed in 11 out of 18 animals in group M. The animals in groups MD_1_-MD_4_ showed decreases in vacuolar degeneration and lymphocytic infiltration in hepatocytes with increasing RE dosages. Over 67% of the animals in both groups MD_1_ and MD_2_ exhibited notably alleviated fibrosis of the liver envelope, portal area, and/or stroma, whereas 67% of the animals in the MD_3_ group and 88% of the animals in the MD_4_ group exhibited obvious aggravation of fibrosis. Typical histopathologic changes are depicted in Fig. 2.

### Biochemical indices

The biochemical indices that changed significantly in the study groups are shown in Tables 1 and 2.

In the control group (N) and RE-treated normal rat groups (ND_1_-ND_4_), the indices exhibited overall dose-dependent increasing trends (Table 1). Compared to the group N, serum ALT and HA values increased significantly in the ND_2_, ND_3_ and ND_4_ groups (*P*<0.05) and AST, LN and TGF-β_1_ values increased significantly in the ND_3_ and ND_4_ groups (*P*<0.05), whereas TBIL values increased significantly in the ND_4_ group only (*P*<0.05).

The serum ALT and AST values of all RE-treated model groups (MD_1_-MD_4_) were significantly lower than those of group M without treatment (*P*<0.05). However, these two indices stopped declining and started to increase when the dosage increased from the second dosage (MD_2_, 5.40 g/kg) to the third (MD_3_, 14.69 g/kg). Regarding the TBIL, HA, LN and TGF-β_1_ levels, the values of the maximum dosage group (MD_4_ group) were even higher than those of group M without treatment (*P*<0.05) (Table 2).

### Factor analysis using biochemical indices as variables

**Figure 3:**
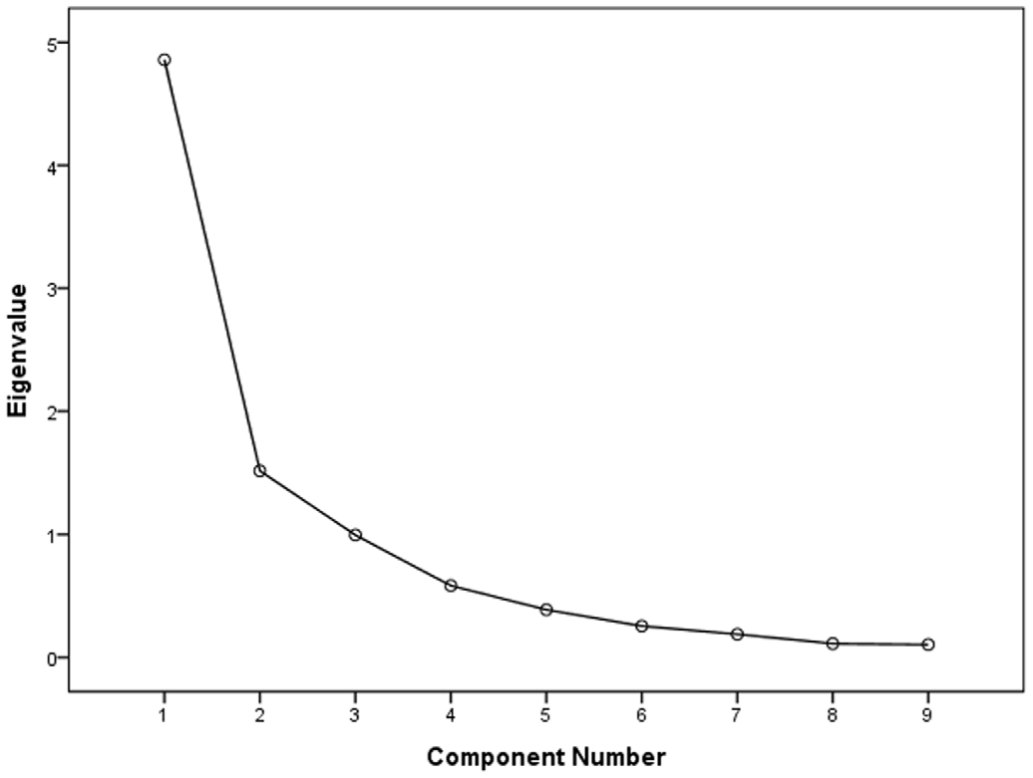
Scree plot depicting the eigenvalues of the factors extracted by factor analysis using biochemical indices as variables. The two factors with eigenvalue greater than 1.0 were retained in subsequent analyses.

Bartlett's test of sphericity indicated a correlation between the currently used variables because the correlation matrix was significantly different from the identity matrix (*P*<0.01). The KMO measure of sampling adequacy was 0.789, which was higher than the acceptable criterion of 0.5. [26] The eigenvalues of the 9 factors are depicted in a scree plot in Fig. 3. Several methods were used to determine the number of meaningful factors that should be extracted. As a general rule, factors with an eigenvalue greater than 1.0 are retained. [27] The rationale for this rule is that the amount of variance accounted for by each factor should be at least equal to the variance of one variable. [24] This criterion indicated that two factors should be extracted in the present study. The first and second factors accounted for 53.985% and 16.856% of the total variability, respectively. These two primary factors could explain 70.840% of the total variability in the dataset. The results of the factor analysis and a suggested interpretation are shown in Table 3. In interpreting the retained factors, we only selected variables with loadings above 0.5 or below -0.5 (i.e., variables with high influence).[28] According to this criterion, factor 1 could be labeled as “fibrosis” because the selected biochemical variables including HA, LN and TGF-β_1_ (all loadings >0.85) are related to the fibrotic course[29]-[31] and factor 2 could be labeled as “cellular injury” because it was mainly affected by ALT and AST (both loadings >0.90), which were altered when the hepatocellular membrane was damaged.

**Table 3:** Results of factor analysis after a varimax rotationa.

Variable	Factor 1	Factor 2
AST	0.068	**0.910**
ALT	0.090	**0.901**
ALP	0.348	**0.620**
TP	0.448	**0.514**
GLO	**0.526**	**0.675**
TBIL	**0.685**	0.434
HA	**0.856**	0.264
TGF-β_1_	**0.856**	0.121
LN	**0.896**	0.103
Eigenvalue	4.859	1.517
Cumulative proportion b	53.985	70.840
Suggested interpretation	Fibrosis	Cellular injury

ALT, serum alanine aminotransferase; AST, serum aspartate aminotransferase; AP, alkaline phosphatase; TBIL, total bile acids; TP, total protein; GLO, globulin; HA, hyaluronic acid; LN, laminin; TGF-β_1_, transforming growth factor β_1_.

a The loadings, eigenvalues, cumulative proportion of the total variance and a suggested interpretation are provided for the two factors. Only variables with loadings above 0.5 or below -0.5 were used in the interpretation (displayed in bold).

b The cumulative proportion is the cumulative proportion of the total variance in the data. Because the variables are standardized to a variance of 1, the total eigenvalue is equal to 9 (number of variables).

**Figure 4:**
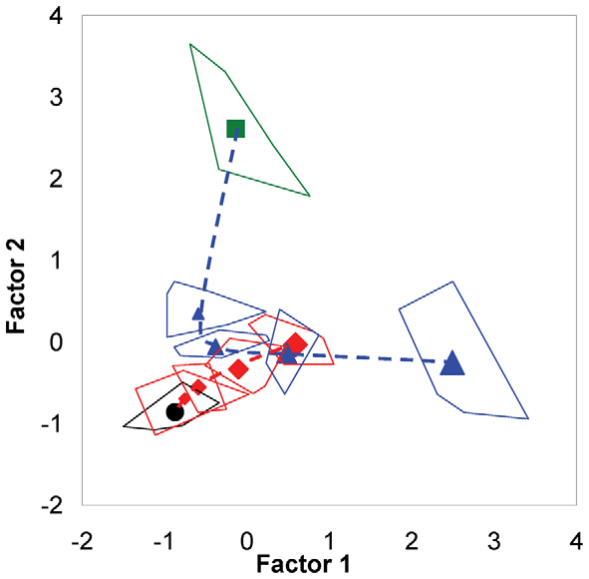
Dose-response relationships of rhubarb extract to both normal and model rats, as represented by a factor plot. The alteration pattern of the dose-response relationship between rhubarb extract and hepatic health in normal rats showed an almost linear variation: factor 1, considered in relation to fibrosis and called the “fibrosis factor”, showed a notable increasing trend with increasing of dosage levels (ND_1_-ND_4_, dosages of 2.00, 5.40, 14.69 and 40.00 g kg^−1^, respectively), whereas factor 2, considered in relation to cell membrane integrality and called the “cellular injury factor”, revealed a nonsignificant increase. By contrast, the alteration pattern in the model rat groups assumed a parabolic shape with increasing dosages. The factor 1 values decreased in the MD_1_ and MD_2_ groups but decreased to a small degree when the dosage was further increased. The factor 2 values decreased slightly in the MD_1_ and MD_2_ groups and then shifted to a significant increasing trend with increasing dosages in the MD_3_ and MD_4_ groups. The center points of the different groups are labeled as follows: untreated normal rats group (N, ▪ green square); normal rat groups treated with rhubarb extract at different dosages (ND_1_-ND_4_, ⧫) (symbol size indicates dosage); untreated model rat group (M, •); and model rat groups treated with rhubarb extract at different dosages (MD_1_-MD_4_, ▴ blue triangle) (symbol size indicates dosage). The border lines around the center points are presented for the range of original data points of the studied groups.

The graphical representation of the dose-response relationship of RE, using these two statistically obtained factors as the axes to show the distribution pattern of different study groups in the plot, is depicted in Fig. 4. In the graphical representation, the toxicity- and pharmacologically induced variation patterns of the biochemical indices could be deduced from the ordered distribution of the studied groups. The untreated N group and the untreated M group are represented at the left lower side and upper right side of the diagram, respectively. The distance of group M away from group N illustrated certain hepatotoxic effects of both enzyme variations and fibrosis in rats with CCl_4_-induced hepatitis compared to normal animals. The values of the fibrosis factor of the normal rat groups increased in all dosage groups (ND_1_-ND_4_, dosages from 2.00 to 40 g/kg), whereas their values of the cellular injury factor did not significantly increase. In the model rat groups, the values of the cellular injury factor of the first (MD_1_, 2.00 g/kg) and second (MD_2_, 5.40 g/kg) dosage groups decreased compared to the value of the untreated model group (M), whereas the values decreased by a smaller degree when the dosage was further increased. Although the values of the fibrosis factor of the MD_1_ and MD_2_ groups decreased slightly compared to the value of the M group, the values of the MD_3_ (14.69 g/kg) and MD_4_ (40.00 g/kg) groups shifted to an increasing trend with increasing dosage.

## Discussion

It has been previously reported that the protective effect of rhubarb against CCl_4_-induced liver lesions was largely attributed to the inhibition of the generation of free radicals and antioxidant activity. [32], [33] The mechanism of CCl_4_-induced liver injury is generally interpreted as follows: the compound is bioactivated by cytochrome P450 2E1 to the trichloromethyl free radical (•CCl_3_) and then further converted to a peroxy radical (CCl_3_O_2_•), which leads to a chain reaction auto-oxidation of the fatty acids in the cytoplasmic membrane phospholipids causing functional and morphological changes to the cell membrane. [34] As a consequence, the cell membranes of hepatocytes become more permeable, and enzymes such as ALT and AST can leak out into the bloodstream, leading to increased levels of these enzymes in the blood. These increases reflect the degree of hepatocyte damage and necrosis [35] and vice versa. Therefore, it could be inferred that the pharmacological activity of rhubarb in treating liver injury is strongly related to its antioxidant effects via the protection of hepatic cells against free radical damage. This outcome manifested as a decrease in the cellular injury factor, (shown in Fig. 4), which was closely correlated with biomarkers such as ALT and AST (shown in Table 2).

The anti-oxidative effects [36] of rhubarb anthraquinone derivatives, along with their hepatoprotective effects, [11] have been reported. In addition, a series of tannin-related compounds with strong antioxidant effects have been identified in RE (see Supplementary Materials). In a previous report, the antioxidant activities of rhubarb tannins were verified. [37] Therefore, we believe that rhubarb tannins and anthraquinone derivatives may all contribute to the hepatoprotective effects of rhubarb against CCl_4_-induced liver damage.

The hepatoprotective effect of rhubarb and its components has also been documented as antagonizing α-naphthylisothiocyanate (ANIT)-[38] and concanavalin A [39]- induced experimental liver injury. In a previous study, we found that free anthraquinones extracted from rhubarb, such as rhein and emodin, exhibited protective activity against ANIT-induced cholestatic liver injury by reducing the serum levels of glutamate-pyruvate transaminase, glutamic oxaloacetic transaminase and the serum total bilirubin, direct bilirubin, alkaline phosphatase, γ-glutamyltransferase and total bile acids. These effects were markedly different from the effects on CCl_4_-induced liver injury. The morphological alterations induced by ANIT in rats, including the necrosis of hepatocytes and biliary epithelial cells, as well as neutrophil infiltration and sinusoid congestion, were also alleviated by concurrent intragastric administration of these free anthraquinones. [8] Some clinical evidence has also revealed a hepatoprotective effect of rhubarb against infantile cholestatic hepatitis[40] and acute icteric hepatitis [41]. The protective effect against CCl_4_-induced liver damage reported in this study demonstrated only one aspect of the pharmacological potential of rhubarb to protect the liver. Although there is some evidence of the protective potential of this herb against liver injury due to multiple causes, the molecular mechanisms of this protection remain unclear. Rigorous clinical studies and in-depth experimental studies are needed to demonstrate rhubarb's hepatoprotective effects and mechanisms of action.

In our study, dose-dependent alterations of liver fibrosis associated with increases in the fibrosis factor were clearly observed in the rats in the normal groups (Figs 2 and 4). Although the occurrence of fibrosis of the liver tissues of normal rats that were treated with rhubarb has been reported infrequently, this phenomenon warrants further consideration. Elevation of TBIL, TP and GLO levels in serum have also been found in normal rat groups. These alterations usually occur along with hepatic impairment, indicating functional deterioration of the liver. Although the hepatoprotective effects of anthraquinone derivatives are well documented, it has also been reported that the total anthraquinone derivatives isolated from rhubarb show some hepatotoxic potential in rats in a six month-long experiment. [14] Therefore, the dose-response relationship between rhubarb anthraquinones and liver health warrants further investigation.

The alteration pattern of the fibrosis factor in the model rat groups assumed a parabolic shape with increasing dosages of RE (shown in Fig. 4). At lower dosages (MD_1_, 2.00 g/kg and MD_2_, 5.40 g/kg), rhubarb demonstrated some ameliorative effects on liver injury by decreasing the fibrosis caused by CCl_4_; however, the fibrosis was aggravated rather than alleviated when the dosage was increased further (MD_3_, 14.69 g/kg and MD_4_, 40.00 g/kg). Therefore, we believe that the hepatotoxic potential of rhubarb is strongly related to liver fibrosis, although this effect is undetectable when the normal dosage recommended in the Chinese Pharmacopoeia (0.5 g/kg) is administered. [20]

The results of the factor analysis of biochemical indices indicated that the parallel histopathological changes arose coincidently. The vacuolar degeneration, and lymphocyte infiltration and fibrosis observed in the hepatocytes of CCl_4_-treated rats were alleviated after treatment with RE, demonstrating the herb's effectiveness in protecting hepatocytes from the toxicant. However, the anti-fibrotic effect of RE that was observed in the lower dosage groups was reversed with further increases in the RE dosage. The dose-dependent hyperplasia of interstitial fibrous tissue was also observed in liver tissue sections from normal rats that were treated with RE, which was in accordance with the variations in the fibrosis factor.

A classical description of the understanding of such bidirectional effects of rhubarb and other herbal medicines, *You Gu Wu Yun*, was recorded in the Chinese Medical Treatise *Su Wen* (translated as *Plain Questions*, part of *The Yellow Emperor's Classic of Internal Medicine*), which was written 2,500 years ago.[42] The archaic and recondite wording of *You Gu Wu Yun* can be explained as follows. A drug will reveal its therapeutic effect when it is prescribed to patients with the correct indications; however, it may produce deleterious effects in both sick and healthy people as a result of incorrect indications. Another translation of the wording implies that the dosage of the drug is critical to its rational application in the clinic to balance the benefits and risks. Ancient Traditional Chinese Medicine (TCM) has effectively relied on the theory of *You Gu Wu Yun* as an important set of guidelines in the treatment of diseases and disorders. The dose-related bidirectional effect of TCM illustrated in this study supports the aforementioned theory. The pattern recognition approach proposed in this report opens an avenue to decode the ancient TCM theory and to elucidate its medical implications with the use of modern biochemical tools.

In conclusion, the findings of this study illustrate the bidirectional potential, both liver protection and hepatotoxicity, of rhubarb on CCl_4_-treated and normal rats and demonstrate the feasibility of using a multivariate analysis approach factor analysis, to study the dose-response (therapeutic efficacy versus toxicity) relationships of traditional herbal medicines by revealing the underlying interrelationships within a number of functional bio-indices in a holistic manner. This study provides a paradigm for a better understanding and scientific assessment of the benefits and risks of herbal medicines to facilitate the rational clinical administration of these medicines.

## Supporting Information

Figure S1 **Chemical structures of the free anthraquinones in the rhubarb extract.**
(TIF)

Figure S2 **Chemical structures of the tannin-related compounds in the rhubarb extract.**
(TIF)

Table S1 **Identification of the chemical constituents in the rhubarb extract.**
(DOC)

Table S2 **The contents (%) of anthraquinones and tannins in the rhubarb extract.**
(DOC)

Text S1 **UPLC- ESI-MS/MS analysis.**
(DOC)
