# Supplementary material for: Hepatotoxicity or Hepatoprotection? Pattern Recognition for the Paradoxical Effect of the Chinese Herb Rheum palmatum L. in Treating Rat Liver Injury
Source: PLoS One. 2011 Sep 6;6(9):e24498. doi: 10.1371/journal.pone.0024498 (PMC3167848; doi:10.1371/journal.pone.0024498)
Supplement: Table S1 — Identification of the chemical constituents in the rhubarb extract. (DOC) [file pone.0024498.s003.doc]

**Table S1 Identification of the chemical constituents in the rhubarb extract**

| Compound | t_R_(min) | Name | [M–H]^－^ | MS^2^ |
| --- | --- | --- | --- | --- |
| 1 | 3.11 | Galloyl glucose | 331 | [331]: 169, 125 |
| 2 | 4.45 | Unknown |  |  |
| 3 | 5.73 | Glucopyranosyl-galloyl-glucose | 493 | [493]: 313, 169 |
| 4 | 7.26 | Catechin-glucopyranoside | 451 | [451]: 289, 205, 137 |
| 5 | 9.33 | Catechin-glucopyranoside | 451 | [451]: 289, 205, 137 |
| 6 | 17.01 | Unknown | 373 | [373]: 355, 309, 220, 189, 161, 147 |
| 7 | 17.24 | Unknown | 442 | [442]: 415, 348, 309, 162, 147 |
| 8 | 18.45 | Unknown | 478 | [478]: 459, 373, 234, 189 |
| 9 | 19.52 | Aloe emodin-*O*-glucoside | 431 | [431]: 269, 240 |
| 10 | 21.55 | Rhein-*O*-glucoside | 445 | [445]: 283, 239, 211 |
| 11 | 22.15 | Cinnamoyl-*O*-galloyl-glucose | 461 | [461]: 313, 169 |
| 12 | 23.65 | Unknown |  |  |
| 13 | 24.73 | Laccaic acid D *O*-glucoside | 475 | [475]: 313, 269 |
| 14 | 27.32 | Dehydroxylaccaic acid D *O*-glucoside | 459 | [459]: 295, 253, 225 |
| 15 | 28.26 | Chrysophanol-*O*-glucoside | 415 | [415]: 253, 225 |
| 16 | 28.85 | Chrysophanol-*O*-glucoside | 415 | [415]: 253, 225 |
| 17 | 29.38 | Emodin-*O*-glucoside | 431 | [431]: 269, 225 |
| 18 | 29.90 | Unknown | 431 |  |
| 19 | 31.70 | Laccaic acid D | 313 | [313]: 269 |
| 20 | 34.14 | Physcion-*O*-glucoside | 445 | [445]: 283, 240 |
| 21* | 34.93 | Emodin | 269 | [269]: 241, 225 |
| 22 | 36.10 | Acetyl- chrysophanol | 296 | [296]: 253, 225 |
| 23* | 40.60 | Physcion | 283 | [283]: 268, 240 |

* Compounds confirmed by comparing with pure standards.
