# Supplementary material for: Hepatotoxicity or Hepatoprotection? Pattern Recognition for the Paradoxical Effect of the Chinese Herb Rheum palmatum L. in Treating Rat Liver Injury
Source: PLoS One. 2011 Sep 6;6(9):e24498. doi: 10.1371/journal.pone.0024498 (PMC3167848; doi:10.1371/journal.pone.0024498)
Supplement: Table S2 — The contents (%) of anthraquinones and tannins in the rhubarb extract. (DOC) [file pone.0024498.s004.doc]

**Table S2 The contents (%) of anthraquinones and tannins in the rhubarb extract**

|  | Aloe-emodin | Rhein | Emodin | chrysophanol | physcion | Sum |
| --- | --- | --- | --- | --- | --- | --- |
| Total AQs | 0.435 | 1.421 | 0.437 | 0.898 | 0.568 | 3.759 |
| Total tannins |  |  |  |  |  | 9.34 |
