# Supplementary material for: Hepatotoxicity or Hepatoprotection? Pattern Recognition for the Paradoxical Effect of the Chinese Herb Rheum palmatum L. in Treating Rat Liver Injury
Source: PLoS One. 2011 Sep 6;6(9):e24498. doi: 10.1371/journal.pone.0024498 (PMC3167848; doi:10.1371/journal.pone.0024498)
Supplement: Text S1 — UPLC- ESI-MS/MS analysis. (DOC) [file pone.0024498.s005.doc]

Supporting information

***UPLC-*** ***ESI-MS/MS analysis***

**Instruments**

The liquid chromatography analyses were performed using a Waters Acquity system (Waters Corporation, Milford, MA, USA) equipped with binary solvent delivery pump, an auto sampler and UV-Vis detector. The electrospray ionization tandem mass spectrometry (ESI-MS/MS) experiments were conducted using a Waters TQ Detector (Waters Corporation, Milford, MA, USA).

**Ultra performance liquid chromatography (UPLC)**

The chromatographic separation was performed using a Waters Acquity T_3_ column (100 mm×2.1 mm, 1.7 μm). Mobile phase consisted of (A) methanol and (B) 0.1% aqueous formic acid using a gradient program of 3-15% A in 0-10 min, 15-40% in 10-40 min, and 40-65% in 20-45 min. The detector wavelength was set at 280 nm. The injection volume was 5 μl while the column was maintained at 35 °C.

**Electrospray ionization tandem mass spectrometry (ESI-MS/MS)**

ESI-MS/MS experiments were conducted in negative ionization mode. Ultrahigh-purity helium (He) was used as the collision gas and high-purity nitrogen (N_2_) as the nebulizing gas. The MS detector was optimized by injecting a 5 μl/min flow of anthraquinone and catechin standards (0.01 mg/ml in methanol) to obtain maximum intensities of [M–H]¯ ions. The optimized parameters in the negative ion mode were as follows: capillary voltage, 3 kV; cone voltage, -45 V; desolvation gas (N_2_) flow of 600 L·min^−1^; desolvation gas temperature, 350 °C.

**Identification of anthraquinone glycosides in the rhubarb extract**

The anthraquinones (AQs) and their glucosides which were found of the main constituents in the extract of rhubarb were identified by liquid chromatography/tandem mass spectrometry (Table S1). The chemical structures of the free anthraquinones were depicted in Figure S1. The total AQs in the extract were determined after hydrolyzation by hydrochloric acid to decompose the conjunct anthraquinones, since these chemicals of anthraquinone structure revealed important bioactivities in rhubarb. Aloe emodin, rhein, emodin, chrysophanol and physcion, the most important AQs, were quantitated by UPLC using pure standards as references and their contents were 0.435%, 1.421%, 0.437%, 0.898% and 0.568%, respectively (Table S2).

**Identification of tannins in the rhubarb extract**

The identified skeleton types of tannins in rhubarb were glucose gallates and catechin glycosides, summarized in Table S1. The chemical structures of identified tannins were depicted in Figure S2. The total tannins were determined by phosphomolybdium tungstic acid-casein colorimetric method, official stipulated in Chinese Pharmacopoeia, with gallic acid as reference substance. The content of total tannins in the rhubarb extract was 9.34% (Table S2).
